# A Randomized, Double-Blind, Multicenter Clinical Study Comparing the Efficacy and Safety of a Drug Combination of Lopinavir/Ritonavir-Azithromycin, Lopinavir/Ritonavir-Doxycycline, and Azithromycin-Hydroxychloroquine for Patients Diagnosed with Mild to Moderate COVID-19 Infections

**DOI:** 10.1155/2021/6685921

**Published:** 2021-02-09

**Authors:** Purwati, Budiono, Brian Eka Rachman, Yulistiani, Andang Miatmoko, Nasronudin, Soroy Lardo, Yongki Iswandi Purnama, Mafidhatul Laely, Ike Rochmad, Taufik Ismail, Sri Wulandari, Dwi Setyawan, Alfian Nur Rosyid, Herley Windo Setiawan, Prastuti Asta Wulaningrum, Tri Pudy Asmarawati, Erika Marfiani, Shinta Karina Yuniati, Muhammad Rabiul Fuadi, Pepy Dwi Endraswari, Purwaningsih, Eryk Hendrianto, Deya Karsari, Aristika Dinaryanti, Nora Ertanti, Igo Syaiful Ihsan, Disca Sandyakala Purnama, Yuni Indrayani

**Affiliations:** ^1^Stem Cell Research and Development Center, Airlangga University, Campus C UNAIR, Mulyorejo, Mulyosari, Surabaya 60115, Indonesia; ^2^Faculty of Vocational Studies, Airlangga University, Campus B UNAIR, Jl. Dharmawangsa Dalam Selatan, Gubeng, Surabaya 60286, Indonesia; ^3^Faculty of Pharmacy, Airlangga University, Nanizar Zaman Joenoes Building, Campus C UNAIR, Mulyorejo, Mulyosari, Surabaya 60115, Indonesia; ^4^Airlangga University Hospital, Campus C UNAIR, Mulyorejo, Mulyosari, Surabaya 60115, Indonesia; ^5^Gatot Soebroto Army Hospital, Jl. Abdul Rahman Saleh, Senen, Jakarta 10410, Indonesia; ^6^COVID-19 Isolation Center, Lamongan, Indonesia; ^7^Dustira Hospital, Jl. Dustira, Baros, Cimahi Tengah, Cimahi 40521, Indonesia; ^8^Polri Hospital, Jl. Raya Bogor, Kramat Jati, Jakarta 13510, Indonesia

## Abstract

**Background:**

At the present time, COVID-19 vaccines are at the testing stage, and an effective treatment for COVID-19 incorporating appropriate safety measures remains the most significant obstacle to be overcome. A strategic countermeasure is, therefore, urgently required.

**Aim:**

This study aims to evaluate the efficacy and safety of a combination of lopinavir/ritonavir-azithromycin, lopinavir/ritonavir-doxycycline, and azithromycin-hydroxychloroquine used to treat patients with mild to moderate COVID-19 infections. *Setting and Design*. This study was conducted at four different clinical study sites in Indonesia. The subjects gave informed consent for their participation and were confirmed as being COVID-19-positive by means of an RT-PCR test. The present study constituted a randomized, double-blind, and multicenter clinical study of patients diagnosed with mild to moderate COVID-19 infection.

**Materials and Methods:**

Six treatment groups participated in this study: a Control group administered with a 500 mg dose of azithromycin; Group A which received a 200/50 mg dose of lopinavir/ritonavir and 500 mg of azithromycin; Group B treated with a 200/50 mg dose of lopinavir/ritonavir and 200 mg of doxycycline; Group C administered with 200 mg of hydroxychloroquine and 500 mg of azithromycin; Group D which received a 400/100 mg dose of lopinavir/ritonavir and 500 mg of azithromycin; and Group E treated with a 400/100 mg dose of lopinavir/ritonavir and 200 mg of doxycycline.

**Results:**

754 subjects participated in this study: 694 patients (92.4%) who presented mild symptoms and 57 patients (7.6%) classified as suffering from a moderate case of COVID-19. On the third day after treatment, 91.7%–99.2% of the subjects in Groups A–E were confirmed negative by a PCR swab test compared to 26.9% in the Control group. Observation of all groups which experienced a significant decrease in virus load between day 1 and day 7 was undertaken. Other markers, such as CRP and IL-6, were significantly lower in all treatment groups (*p* < 0.05 and *p* < 0.0001) than in the Control group. Furthermore, IL-10 and TNF-*α* levels were significantly elevated in all treatment groups (*p* < 0.0001). The administration of azithromycin to the Control group increased CRP and IL-6 levels, while reduced IL-10 and TNF-*α* on day 7 (*p* < 0.0001) compared with day 1. Decreases in ALT and AST levels were observed in all groups (*p* < 0.0001). There was an increase in creatinine in the serum level of the Control, C, D, and E groups (*p* < 0.05), whereas the BUN level was elevated in all groups (*p* < 0.0001).

**Conclusions:**

The study findings suggest that the administration of lopinavir/ritonavir-doxycycline, lopinavir/ritonavir-azithromycin, and azithromycin-hydroxychloroquine as a dual drug combination produced a significantly rapid PCR conversion rate to negative in three-day treatment of mild to moderate COVID-19 cases. Further studies should involve observation of older patients with severe clinical symptoms in order to collate significant amounts of demographic data.

## 1. Introduction

Since late 2019, a global campaign has been waged against the Coronavirus 2019 (COVID-19) pandemic caused by the severe acute respiratory syndrome (SARS-CoV-2) virus which has infected 70.4 million people and caused 1,599,704 deaths worldwide [1]. Various initiatives have been undertaken in an attempt to eradicate the pandemic. However, to date, all efforts to halt the transmission and spread of COVID-19 have proved unsuccessful.

Several studies have reported that the majority of individuals (80%) infected with COVID-19 have presented mild to moderate symptoms [2–4]. Indonesia is densely populated with 270 million inhabitants, the fourth-largest national population in the world. Consequently, the ongoing pandemic has significantly impacted the country in various sectors, including economy, education, and health (source: http://www.covid19.gov.id). The majority of COVID-19 patients in Indonesia fall within productive age ranges, with an average isolation period between 10 and 14 days, factors which have had a significant negative effect on the economic sector. On the other hand, approximately 35% of individuals falling within the country's productive age range live with their parents who are, consequently, identified as a high-risk group in relation to COVID-19 (source: Statistics Indonesia. http://www.bps.go.id). Therefore, proactive initiatives are required to facilitate the prompt social reintegration of those COVID-19 patients presenting mild and moderate symptoms.

Experts around the world have been working unstintingly to find an effective cure for COVID-19. Various drugs such as azithromycin [5], hydroxychloroquine [5, 6], lopinavir/ritonavir [7], remdesivir [7], homoharringtonine [7], and emetine [7] have been reported as demonstrating antiviral potential during preclinical trials. Most represent newly determined indicative uses of previous drug regimens. In Indonesia, researchers have identified several drugs such as lopinavir/ritonavir, azithromycin, doxycycline, and hydroxychloroquine as potentially having curative effects against COVID-19 infection. In a previous study undertaken by the authors of this article, the CC50 values observed for an *in vitro* cytotoxicity assay on mesenchymal cells indicated that a combination of these drugs had a lower degree of toxicity than that of a single drug (unpublished data). The drug combination employed during the in vitro study proved effective in lowering the viral copy numbers in the Vero cells infected with SARS-CoV-2 which had been isolated from hospitalized patients at 72, 48, and, even, 24 hours after drug incubation (unpublished data). Moreover, the research in question also highlighted certain new combination drugs such as lopinavir/ritonavir and azithromycin, lopinavir/ritonavir and doxycycline. Hydroxychloroquine and azithromycin produced higher efficacy in inhibiting and eradicating the SARS-CoV-2 virus than their single form (unpublished data).

Several other recent studies have reported the efficacy and safety of some single drug [5, 6, 7, 8] or other drug combinations [8, 9]. However, many variations permeate their results. The present study evaluated the efficacy and safety of combinations of lopinavir/ritonavir and azithromycin; lopinavir/ritonavir and doxycycline; and azithromycin and hydroxychloroquine for patients suffering from mild to moderate COVID-19 who are undergoing treatment not involving the use of a ventilator.

## 2. Methods

### 2.1. Study Conduct

This study constituted a multicenter, double-blind, and randomized controlled clinical trial (RCT) conducted between July and August 2020 at four research sites in Indonesia. The Ethics Committee granted ethical approval for all centers conducting clinical trial protocols (Persetujuan Pelaksanaan Uji Klinik, PPUK) (No. PP.01.01.1.3.07.20.06) issued by the Indonesian Food and Drugs Administration (Badan Pengawas Obat dan Makanan Republik Indonesia) with an additional letter of approval from the National Institute of Health Research and Development, Indonesian Ministry of Health (Balitbangkes Kementerian Kesehatan RI), and ethical approval no. 159/KEP/2020 issued by the Ethics Committee of Universitas Airlangga Hospital (RS UNAIR).

### 2.2. Research Population

**Figure 1:**
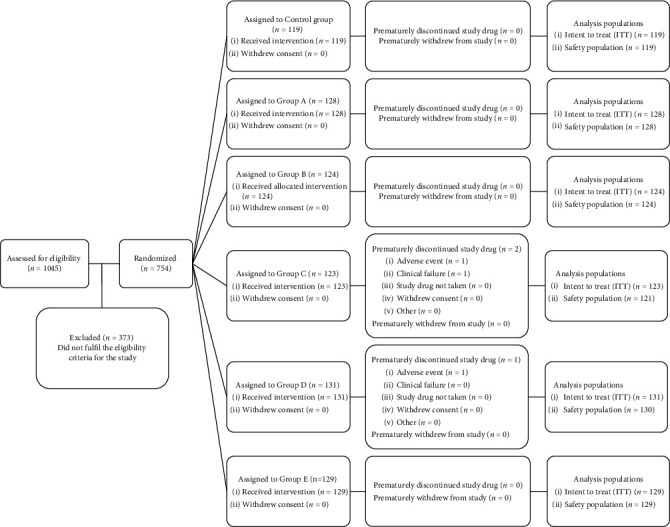
Patients' clinical study disposition algorithm for comparing the efficacy of lopinavir/ritonavir and azithromycin, lopinavir/ritonavir and doxycycline, and hydroxychloroquine and azithromycin drug combinations in improving clinical outcomes of COVID-19 patients hospitalized with mild and moderate symptoms. Control group: 1 × 500 mg azithromycin per day; Group A: 2 × 200/50 mg lopinavir/ritonavir + 1 × 500 mg azithromycin per day; Group B: 2 × 200/50 mg lopinavir/ritonavir + 2 × 100 mg doxycycline per day; Group C: 2 × 100 mg hydroxychloroquine + 1 × 500 mg azithromycin per day; Group D: 2 × 400/100 mg lopinavir/ritonavir + 1 × 500 mg azithromycin per day; Group E: 2 × 400/100 mg lopinavir/ritonavir + 2 × 100 mg doxycycline per day.

For the purposes of this study, 1,045 subjects from four study sites, namely, Universitas Airlangga Hospital (RSUA), Surabaya; Dustira Hospital, Bandung; COVID-19 Isolation Center, Lamongan; and POLRI Hospital, Jakarta, were initially assessed for eligibility before being screened and enrolled in accordance with the inclusion criteria of being male or female adults over the age of 18. The screening process produced 754 eligible subjects who were further randomized into six groups for the purposes of the intervention study as shown in Figure 1. The subjects registering a positive result on the COVID-19 PCR swab test presented mild, moderate, or severe symptoms. Those individuals willing to give informed consent prior to the study were then admitted as patients in one of the closest available hospitals or isolation centers. The exclusion criteria applied to the research subjects comprised the following: pregnant or breastfeeding mothers, individuals with severe liver disorders (indicated by increases in transaminases levels three times or more in excess of the normal range), impaired renal functions (indicated by decreases in creatinine clearance of less than 60 mL/minute), arrhythmia, and/or a compromised potassium/magnesium balance. Moreover, individuals receiving conventional plasma therapy and/or anti-IL-6 therapy who experienced QT prolongation when QTc >60 ms, QTc >500 ms with a narrow QRS, or QTc ³550 ms with wide QRS occurring during treatment or who demonstrated proven resistance to one of the combinations of antibiotics studied, drug allergy events, and adverse events due to the administration of other drugs were excluded from the study (as did those who discontinued their participation).

### 2.3. Randomization and Intervention

The subjects signed an informed consent form confirming their willingness to participate in the study, after which they received the same treatment based on their clinical conditions. Randomized subjects were assigned to one of six treatment groups. The Control group was treated in accordance with the standard of care (SoC), including the administration of 500 mg azithromycin once a day, supplements, and other drugs intended to address clinical symptoms. Group A consisted of subjects treated with a combination of 200/50 mg lopinavir/ritonavir twice a day and 500 mg azithromycin once a day. Group B included subjects treated with a combination of 200/50 mg lopinavir/ritonavir and 100 mg doxycycline twice a day. Group C contained subjects who received 200 mg hydroxychloroquine twice a day and 500 mg azithromycin once a day. Groups D and E were similar to Groups A and B, except that their subjects received a higher dose of 400/100 mg lopinavir/ritonavir twice a day. All groups also received supportive symptom-based treatments.

### 2.4. Study Evaluation: Schedule of Treatments and Evaluation of Study Endpoints

The subjects were administered drugs, received supportive treatment, and underwent physical health monitoring for 7–14 days to evaluate the study. Moreover, the assessed clinical signs were used to assess drug efficacy. An evaluation drawing on a combination of physical examination, clinical radiology, laboratory parameters, and RT-PCR for viral load was also conducted. Any adverse events, serious or otherwise, occurring during the study period were recorded.

The primary objectives of this study were to measure the efficacy of the drug combinations lopinavir/ritonavir and azithromycin; lopinavir/ritonavir and doxycycline; and hydroxychloroquine and azithromycin in improving the clinical outcomes of those COVID-19 patients hospitalized with mild and moderate symptoms. The clinical outcome parameters consisted of improvements in such physical functions as maintaining optimum body temperature (<37.5oC); respiratory rate (≤20 times per minute without the use of auxiliary respiratory muscles); oxygen saturation/SpO_2_ (>95% without provision of supplemental oxygen); and hemodynamic stability (mean arterial pressure/MAP >65 mmHg). Moreover, the decrease in mortality rate was noted to establish the efficacy of drug combination therapy.

The secondary efficacy endpoint was to determine the safety of those drug combinations administered during the study which enhanced the clinical outcomes of COVID-19 patients with mild to moderate symptoms. It also evaluated patient complaints or discomfort, including fever, coughing, breathlessness, sniffles, a sore throat, and other symptoms. Observations were also made using lung X-rays, clinical hematology test results, and cytokine levels, the latter of which were analyzed for IL-2, IL-6, IL-10, and TNF- *α* by means of a Sandwich ELISA method including the use of BT-Labs reagent kits Cat. No. E0094Hu, Cat. No. E0090Hu, Cat. No. E0102Hu, and Cat. No. E0082Hu purchased from the Bioassay Technology Laboratory, China. The viral load was analyzed through quantitative real-time polymerase chain reaction (qRT-PCR), all assays of which were performed using an Applied Biosystems (AP) 7500 Fast Real-Time PCR system (Enigma, Applied Biosystems, Foster city, CA, USA), with Allplex 2019-nCoV Assay PCR reagent (Cat. No. RP10250X, Seegene, South Korea) and a Tiangen extraction kit (Cat. No. DP315-T8, Beijing, China). Viral load analysis was undertaken by first measuring the positive control virus concentration and cycle threshold (Ct) values, with a Qubit fluorometer (Thermo Fisher Scientific, USA). The positive control was an Allplex 2019-nCoV assay kit. The Ct value was converted into copy viral DNA/*μ*l by plotting it as a linearity curve prepared at 8 concentrations.

### 2.5. Statistical Analysis

The 754 subjects were randomized into seven groups constituting six treatment groups and one Control group whose members received SoC. The primary efficacy data were analyzed through head-to-head SoC comparison of treatment groups by means of statistical analysis. Despite there being more than 30 patients in each group, the numerical data (ratio or interval) were further analyzed for normal distribution through the use of a Kolmogorov–Smirnov test. If the data distribution was normal (*p* value ≥0.05), it was further subjected to an analysis of variance (ANOVA) and a least square difference multiple comparison test. However, the data distribution in this study was not normal, leading to further analysis by the administration of Kruskal–Wallis and Mann–Whitney multiple comparison tests. The resulting categorical data were evaluated using a Chi-square test. Moreover, the study of secondary efficacy data used for prestudy and poststudy evaluation of clinical outcome indicators included lung X-rays, laboratory results, and viral load tests.

## 3. Results

### 3.1. Patient Demographics

Of the 1,045 study subjects, 754 were enrolled according to the eligibility criteria shown in Figure 1. The 119 Control group members received a single dose of azithromycin. The 128 Group A patients were administered 200/50 mg lopinavir/ritonavir +500 mg azithromycin. The 124 Group B patients received 200/50 mg lopinavir/ritonavir +100 mg doxycycline. The 123 Group C patients were given a combination of 200 mg hydroxychloroquine and 500 mg azithromycin. The 131 Group D patients were treated with a combination of 400/100 mg lopinavir/ritonavir and 500 mg azithromycin. The medication administered to 129 patients of Group E consisted of 400/100 mg lopinavir/ritonavir + 100 mg doxycycline. Two patients of Group C experienced adverse events during the study and deteriorated clinically causing the researchers to exclude them from further participation in the study. Furthermore, a Group D patient suffered from severe nausea and vomiting, resulting in the immediate termination of the treatment and the removal of that individual from the study.

### 3.2. Evaluation of the Clinical Efficacy of Drug Combination Therapy for COVID-19

Of the 751 study subjects given in Table 1, 694 (92.4%) suffered from mild disease, while 57 (7.6%) presented moderate symptoms. The analysis focused only on the mild symptom group in order to avoid bias. 716 of the research subjects were male (95.3%), while females accounted for only 4.7% (35). Gender was evenly distributed across all treatment groups as confirmed by the Chi-square test results which showed no significant difference (*p* > 0.05). The age range of participants enrolled in this study was between 20 and 55, with a median age of 36-37. A Mann–Whitney study indicated a substantial difference between the Control and D groups and the Control and Groups B, C, and E; however, they were in a close range. The laboratory data results showed that the AST, ALT, serum creatinine, BUN, CRP, and D-dimer values were relatively equal in the Control and A–E groups.

Clinical improvement was assessed on the basis of several symptoms such as fever, sore throat, cough, cold/sniffle, inability to breathe, chest pains/breathlessness, and diarrhea. On day 3, a number of participants continued to experience clinical discomfort, namely, 22 patients in the Control group (18.5%) and 5 subjects (3.9%), 9 subjects (7.3%), 11 subjects (9.1%), 12 subjects (9.2%), and 6 subjects (4.7%), respectively, in Groups A, B, C, D, and E. According to these data, all forms of discomfort had been relieved on the fourth day of treatment.

A report was produced regarding the increase in the D-dimer value related to a poor prognosis, resulting in thrombosis, bleeding, and mortality. This research, therefore, contains an analysis of D-dimer. Based on the data contained in Table 2, a deterioration in the D-dimer rate occurred in all posttherapy groups. No significant difference exists between the Control group and the drug treatment groups. This study also evaluated the CRP rate. The data contained in Table 2 indicate that the CRP value of the Control group and treatment groups A–E ranged from 1.0 to 2.0 on D-1 before experiencing a significant decrease (*P* < 0.0001) to a value of <1.0 on D-7 due to the administration of medication.

To analyze the effectiveness, cytokine levels in the blood including IL-6, IL-10, and TNF-*α* were analyzed on days 1 and 7. On initial examination of the subjects, most of the IL-6 rate values had increased compared to the normal rate in the range of values 7.8–22,022.3 pg/ml with a cutoff point of 9.16 pg/ml in the median value as shown in Table 2. After administering a combination of drug therapies for seven days, an improvement in the Il-6 rate was recorded from a median value of 167.9 ng/ml to one of 186.7 ng/ml (*p* < 0.0001). In Groups A–E, a decrease in the IL-6 rate occurred. In Group A, the median value of 191.0 ng/ml became one of 146.9 ng/ml (*p* < 0.0001); in Group B, the median value of 183.2 ng/ml fell to 145.8 ng/ml (*p* < 0.0001); in Group C, the median value decreased from 180.4 ng/ml to 145.5 ng/ml (*p* < 0.0001); in Group D, the median value fell from 194.2 ng/ml to 170.1 ng/ml (*p* < 0.0001); and in Group E, there was a decrease in the median value from 190.7 ng/ml to 144.2 ng/ml (*p* < 0.0001). These results indicated a significant difference (*p* < 0.0001) between the Control group and the A–E combination drug groups.

The interleukin-10 (IL-10) rate was also monitored. IL-10 is an anti-inflammatory cytokine found in humans whose IL-10 gene encodes IL-10. In this research, there was a mild to moderate increase in IL-10 levels in subjects with a cutoff point of 25.66 pg/ml at the outset of the examination. IL-10 levels ranged from 30.9 to 1,702.9 pg/ml with median values, as listed in Table 2. After seven days of therapy, the results showed that the SoC group demonstrated a reduced level of Il-10 from a median of 141.7 ng/ml to 105.9 ng/ml (*p* < 0.0001). In comparison, the treatment groups recorded an increase in Il-10 levels. In Group A, there was a significant increase from a median value of 82.1 ng/ml to 128.6 ng/ml (*p* < 0.0001). In Group B, the IL-10 value increased from a median value of 89.3 ng/ml to 142.0 ng/ml (*p* < 0.0001); in Group C, the median value increased from 86.9 ng/ml to 144.8 ng/ml (*p* < 0.0001); in Group D, the median value 92.1 ng/ml became 145.7 ng/ml (*p* < 0.0001); and in Group E, the median value increased from 76.0 ng/ml to 147.2 ng/ml (*p* < 0.0001). Based on these results, it can be concluded that a significant difference (*p* < 0.0001) existed between the Control and the A–E combination drug groups. IL-10 plays a role in preventing the occurrence of tissue injury. Consequently, the treatment groups had significantly increased levels of the anti-inflammatory cytokines compared to those of the SoC group.

An initial examination of the research subjects indicated an improvement in TNF-*α* levels on the normal level with a minimum value (5.2–2,316.7) pg/ml, a cutoff point of 3.79 pg/ml, and a median value as shown in Table 2. Following the provision of therapy for seven days, the results showed that the Control group had experienced an increase from a median of 149.3 ng/ml to 179.0 ng/ml (*p* < 0.0001). Meanwhile, there was a significant decrease in TNF-*α* levels (*p* < 0.0001) on the seventh day of therapy. Moreover, significant differences (*p* < 0.0001) were also found between the Control group and the combination drug A–E groups.

**Figure 2:**
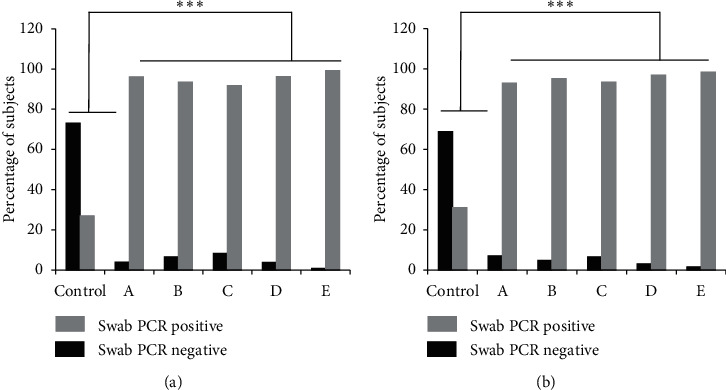
The RT-PCR analysis results of all subjects in the Control and treatment groups of A–E on day 3 (a) and day 7 (b) during the study period (^*∗∗∗*^*p* < 0.0001 compared with the Control). Control group: 1 × 500 mg azithromycin per day; Group A: 2 × 200/50 mg lopinavir/ritonavir + 1 × 500 mg azithromycin per day; Group B: 2 × 200/50 mg lopinavir/ritonavir + 2 × 100 mg doxycycline per day; Group C: 2 × 100 mg hydroxychloroquine + 1 × 500 mg azithromycin per day; Group D: 2 × 400/100 mg lopinavir/ritonavir + 1 × 500 mg azithromycin per day; Group E: 2 × 400/100 mg lopinavir/ritonavir + 2 × 100 mg doxycycline per day.

The RT-PCR analysis on day 3 showed that 26.9% of subjects in the Control group returned a negative result. In contrast, the negative PCR results were in 91.7–99.2% of all the tested subjects observed in Groups A–E. On day 7, there was 31.1% increase in the Control group and about 93.0–98.3% in Groups A–E. A Chi-square analysis revealed a significant difference (*p* < 0.0001) between all tested and Control groups on day 3 and day 7 of treatment, as presented in Figure 2.

In addition to the qualitative analysis conducted, this research undertook quantitative analysis relating to the number of virus copies. There was a significant decrease in this from D-1, D-3, and D-7 in both the SoC and treatment groups. On the other hand, Group E, which was given the usual lopinavir/ritonavir dose, experienced no significant decrease on D-3 or D-7. The pretreatment virus copy inspection results showed that the descending order of groups was that of D, E, Control, C, B, and A. The Kruskal–Wallis test result value was one of *p* < 0.05, indicating a significant pretreatment difference in virus copies. The results of the statistical analysis of observations indicated a tendency for the number of virus copies to have decreased when observed on days 1, 3, and 7, with the median value of the number of virus copies being as listed in Table 3.

### 3.3. Evaluation of Clinical Safety and Tolerability of Drug Combination Therapy for COVID-19

As shown in Table 4, all the treatment groups participating in this study had experienced adverse events. Four Group C subjects complained of headaches, a rapid pulse rate (tachycardia), and pruritus (itchiness) lasting for two days during treatment. Similar symptoms were also observed in four Group A members who experienced headaches for a day, a rapid pulse rate lasting 15 minutes, impaired hearing for a day, and abdominal pain for 30 minutes. Only one Group B subject experienced rapid heartbeat for 15 minutes, while two Group C subjects complained of hearing problems for two days and a rapid heart rate for two hours. Moreover, six subjects experienced diarrhea for one day in addition to headache, nausea, vomiting, abdominal pain, and a rapid pulse rate for 15 minutes. In Group E, three subjects reported experiencing discomfort such as a bitter taste in the mouth, nausea, and abdominal pain for one day. Two Group C subjects dropped out of the study due to severe adverse conditions of a prolonged QT interval >60 ms and clinical failure. The individuals concerned should have started using a ventilator on day 4. Moreover, one Group D subject experienced severe nausea caused by double consumption of antituberculosis drugs, which resulted in his/her withdrawal from the project.

According to the data in Table 4, 24 of the subjects had high leukocyte levels above 12,000 per *μ*L, although there was an improvement (a decrease in the number of leukocytes to within normal limits) on the seventh day. There was no significant difference between the Control group and the A–E drug treatment groups (*p*=0.543). Furthermore, six of the 751 research subjects experienced thrombocytopenia during D-1 therapy. This number was relatively unchanged on D-7, except in Group B which initially contained one person on D-1, subsequently becoming zero on D-7 following treatment. In general, patients did not have lymphocytopenia, with 90–95% of normal patients being on D-1. Only 6–12 of the 751 study subjects experienced lymphocytopenia during D-1 therapy, and this number decreased by half relative to D-7, except in Group B whose members received 200/50 mg of lopinavir/ritonavir and 500 mg of azithromycin combination therapy. Of the 12 individuals on D-1, 11 moved on to D-7 therapy. Overall, there was no significant difference between the Control group and the A–E drug treatment groups.

The research results showed that between 17.7% and 30.5% of research subjects demonstrated high aspartate aminotransferase (AST) levels on D-1. Compared with the Control group, the drug treatment group experienced significantly decreasing numbers between 6.8% and 19.0% as a result of D-7 therapy. Furthermore, as shown in Table 3, SGOT levels decreased significantly between days 1 and 7 in all treatment groups. Furthermore, as many as 164 research sample subjects (21.9%) had pretreatment alanine aminotransferase (ALT) levels above 50 U/L, with this number decreasing to 108 (15.5%) on D-7. Based on the Chi-square test results, no significant difference existed between treatment groups in terms of the number of subjects with ALT levels above 50 U/L either before treatment or on the seventh day after treatment. However, there was an improvement in the liver condition, which was indicated by a significant reduction in ALT levels on day 7 of therapy (*p* < 0.0001) for both treatment and Control groups. Based on these data, nearly a quarter of the total research samples had decreased liver function based on AST and ALT checkup before drug administration. After the 7^th^ day, there was an improvement in liver function. Accordingly, there was no significant difference between AST and ALT in the treatment groups, both before treatment and on the 7th day after treatment.

Furthermore, 29 patients in the study had pretreatment serum creatinine levels above 1.2 mg/dL, a total which increased to 62 on D-7. The Chi-square test results indicated no significant difference after treatment in serum creatinine above 1.2 mg/dL between treatment groups on both D-1 and D-7. Although a statistical increase in the median serum creatinine level occurred, it remained biologically safe. All study samples had BUN levels ≤43 mg/dL, which were still within normal limits on both D-1 and D-7. Paired *t*-test results showed an increase in BUN levels in all treatment groups. However, again, the levels continued to fall within normal limits.

## 4. Discussion

In the present study, the efficacy and safety of drug combination therapies consisting of lopinavir/ritonavir, azithromycin, doxycycline, and hydroxychloroquine were investigated in a randomized, double-blind clinical study design. Several parameters have been determined, including clinical signs and hematological laboratory data comprising blood count, D-dimer, CRP, cytokines profiling, and qualitative and quantitative PCR assays for the virus load to evaluate the efficacy of the drug combinations used in COVID-19 therapy. The safety aspect of the drugs was assessed by observation of clinical discomfort and liver-kidney function test results. The subjects in this study were reasonably distributed in age, ranging between 21 and 55 years, with the majority diagnosed with a mild case of COVID-19. However, the disease progression of COVID-19 increased the mortality rate. Moreover, the disease proved to be both highly contagious and promoting high-risk comorbidity. Therefore, curative action on mild COVID-19 cases constitutes an essential step in preventing the infection from spreading and worsening clinical conditions. Such action also has benefits in terms of reducing the period of self-isolation required for daily work stimulating economic growth.

Treatment groups A–E participating in this study showed improved PCR conversion results on day 3 when 92.9%–98.3% of subjects were confirmed as PCR negative. This figure differed significantly from that of the Control group which had been given azithromycin (*p* < 0.05). This result supports previously reported nonrandomized clinical trials that suggested a combination of several drugs was more effective than individual drugs [10]. However, only a particular type of medications was used to treat severe cases of COVID-19. However, the result of this study did not match that of the clinical trial conducted in China [11]. Moreover, the Chinese patients received lopinavir/ritonavir via a nasogastric tube due to their inability to swallow. Other studies reported that, for such cases, lopinavir/ritonavir would worsen the patient's condition [12].

During the viral copy number or viral load measurement, subgroups other than mild COVID-19 were included since the high rate of recovery in this group enabled rapid positive PCR conversion. The comprehensive analysis showed a significant decrease in subjects with positive PCR for COVID-19 in Groups A to E, which differed significantly from the Control group (*p* < 0.0001). This remarkable discovery revealed that treated groups whose drug combinations contain azithromycin experienced rapid declining rates compared to nonazithromycin groups (day 3 as against day 7). Azithromycin plays a role in rapidly decelerating the process of viral penetration of a cell and as an immune-modulator agent in increasing the production of interferon types I and III [13]. Moreover, azithromycin could activate MDA-5, while RIG-1 genes regulated the viral presentation in cells [14]. The unforeseen result of Group B was a consequence of administering a half dose of lopinavir/ritonavir compared to Group E which experienced a significant decline in viral load. The subanalysis was applied to the group receiving azithromycin combined with hydroxychloroquine (Group C) and resulted in higher viral load declining rates than in the group treated with lopinavir/ritonavir (Group A), 87% and 74.8%, respectively. This result was noteworthy since a previous study had reported that, in mild and moderate COVID-19, a single dose of these drugs produced the opposite effect [15]. Despite using the drug combination therapy, the present investigation involved a larger sample size and several study centers-conducted evaluations.

Hyperinflammatory responses in COVID-19 cases indicated a major decline in the patient's clinical condition. Moreover, the worsening condition was due to the elevation of proinflammatory cytokines levels, i.e., IL-6. Serologically, the IL-6 level increased in COVID-19 patients as their clinical symptoms worsened [16, 17], together with the initial indicator of their cytokine-level fluctuations [18–20]. Other symptoms included an impaired coagulation predictor [21] and severe lung damage [22] necessitating emergency mechanical ventilation [23] and increasing COVID-19 patient mortality [24]. The present study revealed a significant decrease in IL-6 (*p* < 0.0001) on day 7 across all treatment groups (Groups A–E and the Control group). This inconsistency might be due to the role of SARS-CoV-2 in modulating the immune system. As previously reported, the level of IL-6 expression could be activated with other cytokines like TNF-*α* and IL-1*β* [25], as shown in the murine protein model of SARS-CoV-1. This protein has a high structural similarity to SARS-CoV-2 that N (nucleocapsid) protein directly influenced the secretion of IL-6 through NF-*κβ* [26]. The previous discovery was strongly supported by the relation between IL-6 serological level and viral load counts [27]. However, significant variation of IL-10 level was only observed in Groups A and B. Despite its full mechanism remaining unknown, a contradictive result of IL-10 measurement indicated more severity and a higher mortality rate in MERS [28]. The complete opposite is shown in SARS-CoV-1 [29]. The dynamic of IL-10 alteration rates indicates that, as an anti-inflammatory marker, the cytokines level fluctuates in response to a high level of proinflammatory cytokine. Based on this theory, the cytokine level measurement in this study could not depict the dynamic changes during the COVID-19 infection since it had been taken twice during the treatments administered.

Another inflammation indicator used to predict worsening clinical condition in COVID-19 patients is the C-reactive protein (CRP) level [30, 31]. CRP levels decreased significantly on day 7 in all treated groups (Group A–E and the Control group) with a median level of 0.6-0.7 mg/dL which was lower than the cutoff value for high-risk patients (2.69 mg/dL). In this study, the decrease of CRP and D-dimer levels was measured on days 3 and 7. However, there were no significant differences with the Control group (*p* > 0.05) which was probably due to the anti-inflammation effect of azithromycin and doxycycline [32].

Due to the patient's pre-COVID-19 infection medical history, he/she often suffered liver damage as a direct result of the severity of treatment [33, 34]. The condition was worsened by the continued use of hepatotoxic medications such as lopinavir/ritonavir [5, 35]. In this study, a significant rise in the ALT level was observed only in Group C (*p* < 0.05), although no significant clinical effect ensued from the difference (31 mg/dL on day 1 to 35 mg/dL on day 7). Moreover, the prolonged QT interval represented a severe adverse event for hydroxychloroquine-based therapy such as was the case for one subject in Group C. The previous prediction had been based on a toxicity test of mesenchymal cells which reported that the CC_50_ level used in drug combination therapies was lower than that of a single administration of each drug (unpublished data). A kidney function test revealed that the BUN level increased significantly (*p* < 0.05) in all treated groups (Groups A–E and the Control group), although it had no effect on the patient's clinical condition. However, there were no significant differences in any treatment groups other than the Control group, which suggests that every subject experienced different effects during treatments.

During the evaluation, the imbalanced proportional subject distribution and the inadequate analytical laboratory equipment employed at different research sites emerged as the significant drawbacks of this study. Nevertheless, this did not reflect the current condition of hospitals in Indonesia. The broad range of patient symptoms and degree of severity of the disease should be further investigated to enhance current understanding of the benefits of drug combination therapies in relation to the contrasting severity of the disease in COVID-19 patients. The last drawback was due to the upper age limit of subjects being set at 55. This study did not demonstrate the nature of the efficacy of drug combination therapies and drug safety with regard to the geriatric age group.

## 5. Conclusion

The present study confirmed that the proposed combined therapies successfully accelerated the process of PCR negative conversion compared to the Control group which had been administered with azithromycin. Moreover, the inflammation rate decreased on day 7 of the study. Clinical test and liver-kidney function examination results confirmed that the proposed combination of drugs is safe for clinical use. Further studies must be conducted in the near future with older subjects presenting severe symptoms in order to obtain advanced demographic data.

## Figures and Tables

**Table 1:** The baseline physical characteristics and clinical laboratory data of enrolled subjects who completed treatment during the study.

	Total (*n* = 751)	Control	A	B	C	D	E	*p* value
Disease severity^*∗*^								
Mild, *n* (%)		115 (96.6)	120 (93.8)	113 (91.1)	113 (93.4)	117 (90.0)	116 (89.9)	0.303
Moderate, *n* (%)		4 (3.4)	8 (6.3)	11 (8.9)	8 (6.6)	13 (10.0)	13 (10.1)	
Gender^*∗∗*^								
Male	716 (95.3)	113 (95.0)	123 (96.1)	119 (96.0)	118 (97.5)	119 (91.5)	124 (96.1)	
Female	35 (4.7)	6 (5.0)	5 (3.9)	5 (4.0)	3 (2.5)	11 (8.5)	5 (3.9)	0.305
Age (in years)								
Median		37#	37	37#	36#	37	37#	0.105
Minimum		23	32	26	32	21	20	
Maximum		55	49	49	51	55	45	
Laboratory examination								
Median AST (U/L, minimum–maximum)		27 (13–69)	25 (12–78)	26 (12–78)	25 (5–68)	26 (13–65)	26 (13–213)	0.159
Median ALT (U/L, minimum–maximum)		34 (12–144)	32 (16–142)	33 (3–106)	31 (14–141)	36 (11–116)	35 (7–337)	0.317
Median creatinine serum (mg/dL. minimum–maximum)		0.96 ± 0.13	0.94 (0.68–1.34)	0.96 ± 0.12	0.95 (0.77–1.51)	0.98 ± 0.50	0.98 ± 0.12	0.712
BUN level (mg/dL)		10.7 ± 2.2	11.3 ± 1.8	11.1 (6.4–15.8)	11.2 (7.2–18.6)	11.1 ± 2.3	11.1 ± 2.1	
C-reactive protein (CRP)		2.0 (0.1–69.1)	1.5 (0.1–35.2)	1.0 (0.1–32.4)	1.5 (0.1–43.7)	1.0 (0.0–77.3)	1.2 (0.1–65.1)	0.026
D-dimer		203 (99–1,085)	166.5 (73–776)	177 (84–981)	176 (63–18,460)	191 (75–4,474)	180 (53–2,393)	0.078

^*∗*^ *χ* ^2^ = 6.031; ^*∗∗*^*χ*^2^ = 3.952; ^#^significant difference from Group D. Control group: 1 × 500 mg azithromycin per day; Group A: 2 × 200/50 mg lopinavir/ritonavir + 1 × 500 mg azithromycin per day; Group B: 2 × 200/50 mg lopinavir/ritonavir + 2 × 100 mg doxycycline per day; Group C: 2 × 100 mg hydroxychloroquine + 1 × 500 mg azithromycin per day; Group D: 2 × 400/100 mg lopinavir/ritonavir + 1 × 500 mg azithromycin per day; Group E: 2 × 400/100 mg lopinavir/ritonavir + 2 × 100 mg doxycycline per day; AST: aspartate aminotransferase serum; ALT: alanine aminotransferase serum; BUN: blood urea nitrogen; D-dimer: fibrin degradation fragment.

**Table 2:** Analysis of laboratory data profiles of D-dimer, CRP level, interleukins, and TNF-*α* of subjects in the Control group and Groups A–E on day 1 and day 7 during treatment.

Group	Control	A	B	C	D	E	*p* value
Median level of D-dimer (ng/mL FEU, minimum–maximum)							
Day 1	203 (99–1,085)	166.5 (73–776)	177 (0–981)	176 (63–18,460)	191 (75–4,474)	180 (53–2,393)	0.078
Day 7	169 (70–1,309)	158 (66–481)	152 (66–1,156)	160 (56–485)	173 (68–1,842)	161 (60–819)	0.549

CRP level (mg/L, minimum–maximum)							
Day 1	2.0 (0.1–69.1)	1.5 (0.1–35.2)	1,0 (0.1–32.4)	1.5 (0.1–43.7)	1.0 (0.0–77.3)	1.2 (0.1–65.1)	0.026
Day 7	0.7 (0.0–14.2)	0.6 (0.1–41.5)	0.6 (0.1–24.2)	0.6 (0.1–44.4)	0.6 (0.1–18.1)	0.8 (0.1–34.5)	0.039

IL-6 level (ng/mL, minimum–maximum)							
Day 1	167.9 (7.8–500.4)	191.0 (10.2–1,348.9)	183.2 (25.8–2,934.9)	180.4 (13.2–22,022.3)	194.2 (15.7–1,452.2)	190.7 (32.5–1,348.9)	<0.0001
Day 7	186.7^*∗*^ (18.3–2,432.9)	146.9^*∗*^ (0.2–407.1)	145.8^*∗*^ (19.8–1,753.9)	145.5^*∗*^ (6.3–2,940.0)	170.1^*∗*^ (0.4–820.2)	144.2^*∗*^ (3.5–476.7)	<0.0001

IL-10 level (*ρ*g/mL, minimum–maximum)							
Day 1	141.7 (53.7–1,702.9)	82.1 (35.5–342.5)	89.3 (35.5–404.1)	86.9 (30.9–388.8)	92.1 (32.7–408.1)	76.0 (39.3–319.9)	<0.0001
Day 7	105.9^*∗*^ (36.8–396.3)	128.6^*∗*^ (45.1–1,190.9)	142.0^*∗*^ (45.9–2,132.9)	144.8^*∗*^ (48.0–2,132.9)	145.7^*∗*^ (51.5–740.0)	147.2^*∗*^ (62.4–586.0)	<0.0001

Plasma level of TNF-*α* (*ρ*g/mL, minimum–maximum)							
Day 1	149.3 (5.2–821.0)	168.5 (49.9–2,316.7)	176.6 (56.0–872.2)	165.1 (52.9–1,185.5)	171.5 (47.1–1,026.4)	197.7 (59.4–808.6)	<0.0001
Day 7	179.0^*∗*^ (26.0–1,152.2)	137.9^*∗*^ (28.1–622.0)	143.8^*∗*^ (36.3–641.7)	138.6^*∗*^ (31.7–631.8)	142.6^*∗*^ (1.3–593.4)	130.8^*∗*^ (37.4–380.9)	<0.0001

Control group: 1 × 500 mg azithromycin per day; Group A: 2 × 200/50 mg lopinavir/ritonavir + 1 × 500 mg azithromycin per day; Group B: 2 × 200/50 mg lopinavir/ritonavir + 2 × 100 mg doxycycline per day; Group C: 2 × 100 mg hydroxychloroquine + 1 × 500 mg azithromycin per day; Group D: 2 × 400/100 mg lopinavir/ritonavir + 1 × 500 mg azithromycin per day; Group E: 2 × 400/100 mg lopinavir/ritonavir + 2 × 100 mg doxycycline per day; IL-6: interleukin-6; IL-10: interleukin-10; TNF-*α*: tumor necrosis factor-*α*; ^*∗*^*p*=0.0001 compared with day 1.

**Table 3:** The results of the copy number of the virus of Control and treatment groups were analyzed in subjects with mild severity and total subjects evaluated using qRT-PCR on treatment days 1, 3, and 7.

Period of treatment	Control	A	B	C	D	E	*p* value
Copy number of virus in subjects with mild severity							
Day 1	193.2 (14.1–48,113.7)	67.0 (11.9–4,531.4)	73.2 (11.9–2,945.8)	173.3 (12.4–5,116.0)	828.8 (11.8–370,523.6)	588.4 (11.0–11,877.6)	
Day 3	49.9.0 (0.0–15,085.1)	0.0 (0.0–120.6)	0.0 (0.0–172.8)	0.0 (0.0–617.9)	0.0 (0.0–1,341.9)	0.0 (0.0–1,547.8)	
Day 7	19.8 (0.0–1,445.6)	0.0 (0.0–3,191.4)	0.0 (0.0–66.7)	0.0 (0.0–100.7)	0.0 (0.0–114.8)	0.0 (0.0–148.3)	

Copy number of virus in total subjects							
Day 1	193.2 (14.1–48,113.7)	67.0 (11.9–4,531.4)	73.2 (11.9–2,945.8)	183.6 (12.4–5,116.0)	854.8 (11.8–370,523.6)	670.0 (11.0–11,877.6)	0.001
Day 3	45.4 (0.0–15,085.1)	0.0 (0.0–120.6)	0.0 (0.0–172.8)	0.0 (0.0–617.9)	0.0 (0.0–2,900.6)	0.0 (0.0–1,547.8)	0.012
Day 7	19.8 (0.0–1,445.6)	0.0 (0.0–3,191.4)	0.0 (0.0–66.7)	0.0 (0.0–100.7)	0.0 (0.0–278.0)	0.0 (0.0–148.3)	0.039

Control group: 1 × 500 mg azithromycin per day; Group A: 2 × 200/50 mg lopinavir/ritonavir + 1 × 500 mg azithromycin per day; Group B: 2 × 200/50 mg lopinavir/ritonavir + 2 × 100 mg doxycycline per day; Group C: 2 × 100 mg hydroxychloroquine + 1 × 500 mg azithromycin per day; Group D: 2 × 400/100 mg lopinavir/ritonavir + 1 × 500 mg azithromycin per day; Group E: 2 × 400/100 mg lopinavir/ritonavir + 2 × 100 mg doxycycline per day.

**Table 4:** The adverse events observed in the research subjects during the study period.

Adverse events	Number of subjects (*n*)							
		Control	A	B	C	D	E	*p* value
Nausea						1	1	
Vomiting						1		
Dizziness		2	1			1		
Pruritus		1						
Tachycardia		1	1	1	1	1		
Hearing loss			1					
Abdominal pain			1			1	1	
Otalgia					1			
Diarrhea						1		
Taste loss							1	

Number of patients with leukocytosis (platelet count >12,000 per *μ*L), *n* (%)								
Day 1		4 (3.4)	4 (3.1)	4 (3.2)	4 (3.3)	3 (2.3)	5 (3.9)	0.543
Day 7		1 (1.0)	0 (0.0)	2 (1.7)	1 (0.9)	1 (0.9)	2 (1.7)	0.891

Number of patients with thrombocytopenia (platelet count <150,000 per *μ*L), *n* (%)								
Day 1		2 (1.7)	0 (0.0)	1 (0.8)	0 (0.0)	0 (0.0)	3 (2.3)	0.331
Day 7		1 (1.0)	0 (0.0)	0 (0.0)	0 (0.0)	0 (0.0)	3 (2.6)	0.147

Number of patients with lymphocytopenia (lymphocyte count <1,500 per *μ*L), *n* (%)								
Day 1		6 (5.1)	6 (4.7)	12 (9.7)	10 (8.3)	12 (9.2)	10 (7.8)	0.559
Day 7		2 (1.9)	3 (2.4)	11 (9.3)	7 (6.1)	5 (4.4)	7 (6.0)	0.102

Number of patients with an increase of AST level, *n* (%)								
Day 1	>33 U/L	36 (30.5)	31 (24.4)	22 (17.7)	23 (19.0)	31 (23.8)	35 (27.1)	0.168
Day 7	>33 U/L	19 (18.1)	13 (10.2)	8 (6.8)	20 (17.4)	13 (11.4)	22 (19.0)	0.029

Number of patients with an increase of ALT level, *n* (%)								
Day 1	>50 U/L	27 (22.8)	27 (21.3)	22 (15.7)	19 (15.7)	34 (26.2)	35 (27.1)	0.185
Day 7	>50 U/L	21 (20.0)	19 (14.8)	12 (10.2)	21 (18.3)	14 (12.3)	21 (18.1)	0.270

Number of patients categorized according to serum creatinine level, *n* (%)								
Day 1	>1.2 mg/dL	4 (3.4)	6 (4.8)	4 (3.2)	3 (2.5)	7 (5.4)	5 (3.9)	0.860
Day 7	>1.2 mg/dL	9 (8.6)	8 (6.3)	8 (6.8)	11 (9.6)	11 (9.6)	15 (12.9)	0.515

Median level of AST (U/L, min–max)								
Day 1		27 (13–69)	25 (12–78)	26 (12–78)	25 (5–68)	26 (13–65)	26 (13–213)	
Day 7		27 (17–49)^*∗*^	22 (14–55)^*∗∗∗*^	24 (14–58)^*∗∗*^	25 (6–92)^*∗*^	25 (14–53)^*∗*^	25 (16–73)^*∗*^	<0.05^*∗*^

Median level of ALT (U/L, min–max)								
Day 1		34 (12–144)	32 (16–142)	32 (3–106)	31 (14–141)	36 (11–116)	35 (7–337)	
Day 7		33 (16–106)^*∗∗∗*^	26 (12–140)^*∗∗∗*^	28 (2–137)^*∗∗∗*^	35 (14–205)^*∗∗∗*^	32 (2–91)^*∗∗∗*^	28 (8–128)^*∗∗∗*^	<0.05^*∗*^

Median level of creatinine serum (mg/dL, min–max)								
Day 1		0.95 ± 0.12	0.93 (0.68–1.34)	0.96 ± 0.12	0.95 (0.77–1.34)	0.97 ± 0.14	0.97 ± 0.13	
Day 7		0.99 ± 0.16^*∗∗*^	0.94 (0.67–1.37)	0.95 ± 0.13	0.96 (0.71–1.31)^*∗*^	1.02 ± 0.16^*∗∗∗*^	0.99 ± 0.15^*∗∗*^	<0.05^*∗*^

BUN level (mg/dL)								
Day 1		10.7 ± 2.2	11.3 ± 1.8	11.1 (6.4–15.8)	11.2 (7.2–18.6)	11.1 ± 2.3	11.3 ± 2.1	
Day 7		11.6 ± 2.5^*∗∗∗*^	11.9 ± 2.2^*∗∗∗*^	11.7 (7.0–21.3)^*∗∗∗*^	12.0 (8.3–20.1)^*∗∗∗*^	12.4 ± 2.5^*∗∗∗*^	13.0 ± 2.6^*∗∗∗*^	<0.05^*∗*^

Control group: 1 × 500 mg azithromycin per day; Group A: 2 × 200/50 mg lopinavir/ritonavir + 1 × 500 mg azithromycin per day; Group B: 2 × 200/50 mg lopinavir/ritonavir + 2 × 100 mg doxycycline per day; Group C: 2 × 100 mg hydroxychloroquine + 1 × 500 mg azithromycin per day; Group D: 2 × 400/100 mg lopinavir/ritonavir + 1 × 500 mg azithromycin per day; Group E: 2 × 400/100 mg lopinavir/ritonavir + 2 × 100 mg doxycycline per day; AST: aspartate aminotransferase serum; ALT: alanine aminotransferase serum. Normally distributed data presented in mean ± SD was analyzed by means of a paired *t*-test, whereas the other data was analyzed using a Wilcoxon signed-rank test. ^*∗*^*p* < 0.05 compared to day 1, ^*∗∗*^*p* < 0.001 compared to day 1, and ^*∗∗∗*^*p* < 0.0001 compared to day 1.

## Data Availability

The data used to support the findings of this study are included within the article.
